# A randomised, open-label, cross-over clinical study to evaluate the pharmacokinetic, pharmacodynamic and safety and tolerability profiles of tobacco-free oral nicotine pouches relative to cigarettes

**DOI:** 10.1007/s00213-022-06178-6

**Published:** 2022-06-23

**Authors:** Fiona Chapman, Simon McDermott, Kathryn Rudd, Victoria Taverner, Matthew Stevenson, Nveed Chaudhary, Kerstin Reichmann, Joseph Thompson, Thomas Nahde, Grant O’Connell

**Affiliations:** 1 Imperial Brands PLC, 121 Winterstoke Road, Bristol, BS3 2LL UK; 2 Reemtsma Cigarettenfabriken GmbH, an Imperial Brands PLC Company, Albert-Einstein-Ring-7, 22761 Hamburg, Germany

**Keywords:** Tobacco harm reduction, Nicotine pouches, Nicotine, Oral nicotine delivery, Tobacco-free nicotine pouches, Cigarettes, Smoking

## Abstract

**Rationale:**

Tobacco harm reduction (THR) involves encouraging adult smokers who would otherwise continue to smoke to transition to less harmful forms of nicotine delivery. These products must offer adult smokers reduced exposure to chemicals associated with tobacco combustion, satisfactory blood plasma nicotine levels and serve as an acceptable alternative. The most recent THR innovation is tobacco-free oral nicotine pouches.

**Objectives:**

This study aimed to compare pharmacokinetic, pharmacodynamic and safety and tolerability profiles of two nicotine pouch variants (ZoneX #2 (5.8 mg nicotine/pouch); ZoneX #3 (10.1 mg nicotine/pouch)) with cigarette to assess the pouches’ THR potential.

**Methods:**

This was a controlled use, randomised, open-label, cross-over clinical study with 24 healthy adult traditional tobacco users. Pharmacokinetic (plasma nicotine levels; up to 8 h post-use), pharmacodynamic (urge to smoke, product liking; up to 4 h post-use) and short-term safety and tolerability profiles were assessed.

**Results:**

Distinct nicotine pouch pharmacokinetic profiles indicated nicotine absorption via the oral mucosa. Plasma nicotine levels were lower, and time to peak slower, for the nicotine pouches compared to cigarette (*C*_max_ cigarette: 11.6 ng/ml vs. #2: 5.2 ng/ml, *p* < 0.0001; #3: 7.9 ng/ml, *p* < 0.0003) (*T*_max_ cigarette: 8.6 min vs. #2: 26 min; #3: 22 min). All products effectively reduced subjects’ urge to smoke and presented favourable product liking scores; nicotine pouches were also well tolerated following short-term use (no serious adverse events).

**Conclusions:**

Overall, the assessed ZoneX nicotine pouches may offer an acceptable alternative for adult smokers to achieve satisfactory levels of nicotine delivery and, based on the pharmacokinetic parameters and under the study conditions, likely have a lower abuse liability and addictive potential for current adult smokers compared to continued cigarette smoking.

Clinical trial identifier: NCT04891406 (clinicaltrials.gov).

**Supplementary information:**

The online version contains supplementary material available at 10.1007/s00213-022-06178-6.

## Introduction

Smoking is a cause of serious diseases in smokers, including lung cancer, heart disease and emphysema (U.S. 2014). The primary cause of these smoking-related diseases comes from burning tobacco (combustion) and inhaling the smoke that is produced (U.S. 2014; Centers for Disease Control and Prevention US 2010). In addition to nicotine, cigarette smoke contains around 7000 other chemicals, a number of which are known toxicants (Centers for Disease Control and Prevention US 2010; FDA, 2012). Public health experts have concluded that whilst nicotine is an addictive substance and not completely risk free, it is not the primary cause of smoking-related diseases; inhalation of tobacco smoke is (RCP, 2016). Smoking cigarettes is therefore considered the most harmful form of nicotine consumption (Nutt et al., 2014).

Complete cessation of all tobacco and nicotine use is the best course of action adult smokers can take to improve their health (Abrams et al., 2018). However, despite numerous public health campaigns, legislations and behavioural deterrents, millions of adults continue to smoke (O'Leary and Polosa 2020). It is here that the public health concept of tobacco harm reduction (THR) becomes important as a next best option for adult smokers. THR involves transitioning adult smokers, who would otherwise continue to smoke, to other nicotine-containing products likely to be substantially less harmful than smoking tobacco (RCP, 2007; RCP, 2016; McNeill and Munafò 2013). This in turn has the potential to lead to substantial reductions in smoking-related morbidity and mortality across populations (O'Leary and Polosa 2020).

A number of more recent product innovations are available for adult smokers, which have the potential to offer significantly less harmful nicotine delivery, including heated tobacco, traditional tobacco-containing Scandinavian snus, high-quality modern chewing tobacco, modern pouched snus and e-cigarettes (Clarke et al., 2019; McNeill et al., 2018). Use of these products does not involve combustion of tobacco, and this translates directly to the presence of, and exposure to, fewer and substantially lower levels of a number of associated toxicants (Gale et al., 2021; Clarke et al., 2019; Morris 2021; Azzopardi 2021). This reduction in exposure has been shown to translate directly to reduced toxicity and therefore may result in a reduction in the adverse health effects reported with tobacco smoking (Polosa et al., 2019; Polosa et al., 2020; Rudd et al., 2020; Meier et al., 2020; Lee 2013; Clarke et al., 2019; Simonavicius et al., 2019; Jaunky et al., 2018; Yu et al., 2022). Further to this growing recognition and scientific evidence that not all nicotine products are equally as harmful as cigarettes, nicotine delivery products are proposed to sit across a continuum of risk (Nutt et al., 2014; McNeill and Munafò 2013). On this relative risk scale, cigarettes sit at one end (the most harmful nicotine delivery product, associated with the highest risk of developing smoking-related diseases) and medicinal nicotine replacement therapies (NRTs) at the other, with the various non-combustible nicotine delivery innovations (described above) in between (McNeill and Munafò 2013; Nutt et al., 2014; Abrams et al., 2018; Murkett et al., 2020).

One of the most recent nicotine delivery product innovations is tobacco-free oral nicotine pouches (hereafter, nicotine pouches), which are available in an increasing number of countries as a potentially less harmful alternative to continued cigarettes smoking. Typically, nicotine pouches contain high-quality pharmaceutical grade nicotine, derived from tobacco leaf, in a dry powder format or mixed with a plant fibre–based substrate, in addition to other high-quality ingredients: flavourings, humectants to retain moisture content and additives to ensure product stability. Nicotine pouches are placed under the user’s lip, and nicotine is absorbed into the bloodstream via the oral mucosa (Lunell et al., 2020; Rensch et al., 2021; McEwan 2021), unlike smoking tobacco, and inhalation of heated tobacco and e-cigarette aerosols, where nicotine is predominantly absorbed via the airways and lungs (O'Connell et al. 2016). Further to this, in contrast to traditional tobacco-containing Scandinavian snus, which also deliver nicotine orally, nicotine pouches do not contain tobacco leaf.

Due to the absence of tobacco leaf and combustion when using nicotine pouches, it is expected that levels of associated toxicants such as tobacco specific nitrosamines (TSNAs), polycyclic aromatic hydrocarbons (PAHs) and carbon monoxide present will be substantially reduced, if at all present, compared to tobacco-containing or combustible tobacco products (Patwardhan and Fagerström 2021). Further to this, as nicotine pouch use does not involve inhalation, lung-related toxicity and risks, and additionally any potential risk to bystanders, are not to be expected. It is these factors which may place nicotine pouches as potentially lower harm nicotine delivery products than snus, heated tobacco and e-cigarettes, which themselves offer significant harm reduction potential and have had a demonstrable impact on smoking rates (RCP, 2016; McNeill et al., 2018; Clarke et al., 2019; Azzopardi 2021).

There is currently limited but increasing evidence in the scientific literature on the THR potential of nicotine pouches. This includes chemical analyses, which have demonstrated reduced levels of tobacco-leaf and smoke-related toxicants within nicotine pouches (Azzopardi 2021), which in turn translates to reductions in in vitro toxicological effects compared to tobacco-leaf containing comparators (Bishop et al., 2020; East et al., 2021; Yu et al., 2022). Emerging clinical evaluations also demonstrate that nicotine pouches can deliver levels of nicotine to the blood comparable to other smokeless tobacco products (Lunell et al., 2020; McEwan 2021) and to a satisfactory level (Rensch et al., 2021). An important factor in the acceptance of such potentially reduced harm nicotine delivery products by adult smokers is the ability to achieve satisfactory levels of nicotine uptake and absorption. The typically slower and lower levels of blood nicotine delivery with nicotine pouches compared to cigarette smoking result in a reported lower abuse liability potential of these products (Rensch et al., 2021). The limited studies currently available on nicotine pouches often use snus, a proven reduced harm product (Clarke et al., 2019), as the comparator product (East et al., 2021; Bishop et al., 2020; Lunell et al., 2020); however, to gain the most information on the THR potential for adult smokers, direct comparison to tobacco, the most harmful form of nicotine delivery, is considered informative and appropriate.

As mentioned, an important part of the THR potential of alternative, potentially reduced harm products is offering a satisfactory level of nicotine delivery to current adult smokers to enable their acceptance over cigarettes. To this end, we conducted a randomised, open-label, cross-over clinical study with 24 adult users of traditional tobacco products (cigarettes and Scandinavian snus) in a controlled clinical setting. The primary aim of the study was to compare the blood plasma nicotine levels following controlled use of one of three commercially available study products, two ZoneX nicotine pouch variants (5.8 mg and 10.1 mg nicotine/ pouch) and a cigarette comparator to assess any differences in the nicotine pharmacokinetic (PK) profiles over 8 h. The secondary aims of this study were to assess subjects’ satisfaction following use of these products, through their desire to smoke and product liking within a 4-h period following use, i.e. pharmacodynamic data, and to assess the products’ short-term safety and tolerability profiles.

## Methods

### Study design

The randomised, open-label, cross-over, confinement study was approved by the Swedish Ethical Review Authority (SERA), performed in accordance with ethical principles set forth in the Declaration of Helsinki and was compliant with the International Conference of Harmonisation (ICH)/ Good Clinical Practice (GCP), European Union Clinical Trials Directive and applicable local regulatory requirements. Twenty-four male and female adult snus and cigarette consumer subjects participated in this study. The study was performed at a single clinical site, and subjects attended two visits, a screening visit and a 5-day confinement period. A follow-up telephone call was also conducted for each subject within a week of the last product use. All subjects provided written informed consent prior to study commencement. Clinical Trial identifier: NCT04891406 (clinicaltrials.gov).

### Subjects

**Fig. 1:**
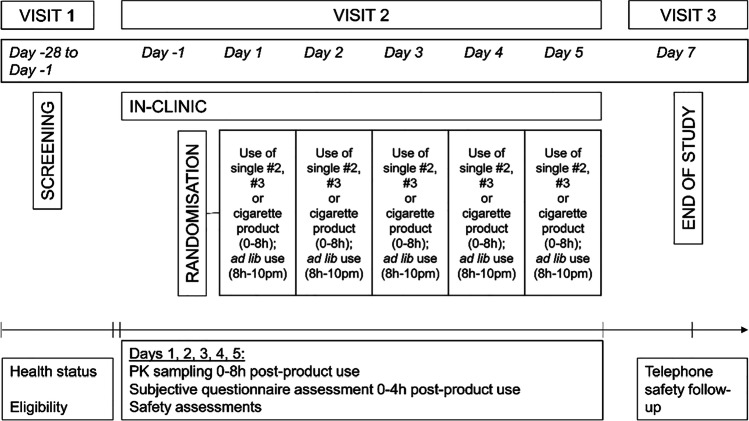
Study design overview, where three products: two nicotine pouches (#2 (5.8 mg nicotine/pouch) and #3 (10.1 mg nicotine/pouch)) and one cigarette product, were randomised to 24 subjects for assessments across 5 days

In total, 42 subjects were screened for participation in the study, and 24 subjects were randomised to the treatment groups (Fig. 1). No formal sample size calculation was carried out for this study; however, the sample size (24 (22 upon completion)) was considered sufficient to provide adequate information in line with the study objectives (i.e. for statistical analyses) and based on similar study designs in the published literature (O'Connell et al. 2016; Rensch et al., 2021). Of the 42 subjects screened, 10 did not meet the eligibility criteria, 6 withdrew consent prior to randomisation, and 2 were reserves not included in the study. Twenty-four subjects started the study, and two withdrew consent, with the reasons stated as private, following the first product use; therefore, 22 subjects attended all study visits and completed the study.

Inclusion criteria for the study were that subjects were willing and able to give written informed consent to partake in the study; were a male or female aged ≥ 19 years at the time of screening; had a BMI of ≥ 18.0 and ≤ 30 kg/m^2^; had a clinically normal medical history, physical findings, vital signs, ECG and laboratory values at the time of screening (evaluated by the Investigator); were a dual user of snus and cigarettes for ≥ 1 year with a minimum weekly consumption of two or more snus cans and > 5 cigarettes and were willing and able to use brands with a nicotine content of ≥ 1%. This was to ensure subjects were well acquainted with the effects of nicotine.

Subjects were not allowed to enter the study if any of the following exclusion criteria were met: a history of any clinically significant disease or disorder which, in the opinion of the investigator, could put the subject at risk because of participation in the study, or influence the results or the subject’s ability to participate in the study; any clinically significant illness, medical/surgical procedure or trauma within 4 weeks of the first use of the study products; any planned major surgery within the duration of the study; any positive result on screening for serum hepatitis B surface antigen, hepatitis C antibody or HIV; after 10-min supine rest at the time of screening, any vital signs values outside of the following ranges: systolic blood pressure < 90 or > 140 mmHg, diastolic blood pressure < 50 or > 90 mmHg, or pulse < 40 or > 90 bpm; pregnant or currently breast feeding female subjects; a history of severe allergy/hypersensitivity or ongoing allergy/hypersensitivity, as judged by the investigator, or a history of hypersensitivity to drugs with a similar chemical structure or class to nicotine; planned treatment or treatment with another product (within 1 month) or investigational drug (within 3 months) prior to day -1 of the study (subjects consented and screened but not dosed in previous phase I studies were not excluded); a positive screen for drugs of abuse or alcohol at screening or on admission to the research unit prior to use of the study products; a history of alcohol abuse or excessive intake of alcohol, as judged by the investigator; presence or history of drug abuse, as judged by the investigator; a history of, or current use of, anabolic steroids, as judged by the investigator; excessive caffeine consumption, defined by a daily intake of > 5 cups of caffeine containing beverages; plasma donation within 1 month of screening or blood donation (or corresponding blood loss) during the 3 months prior to screening; an intention to change their smoking habit or make a quit attempt within 3 months from the screening visit; the Investigator considered the subject unlikely to comply with study procedures, restrictions and requirements.

The participant population consisted of 21 males and 3 females, of whom 22 were of non-Hispanic or Latin ethnicity and 2 were of Hispanic or Latin ethnicity. Twenty-three subjects were white, and one was Asian. The mean age (SD) was 30.4 years (10.0), and the mean BMI (SD) was 24.2 (kg/m^2^3.6).

### Study products

Three test products were used in this study. Two commercially available nicotine pouches were used, ZoneX #2 (5.8 mg nicotine/pouch) (hereafter, nicotine pouch #2) and ZoneX #3 (10.1 mg nicotine/ pouch) (hereafter, nicotine pouch #3) (manufacturer, Skruf Snus, A.B., Sweden, a wholly owned subsidiary of Imperial Brands PLC). These products contain high-purity pharmaceutical-grade nicotine combined with a food-grade plant fibre–based substrate (they do not contain tobacco leaf) and other high-quality ingredients including flavourings, humectants to retain moisture and additives to ensure product stability. Both nicotine pouch products were formulated with the flavour Cold Blast, which has a mint flavour profile. The third product was the Marlboro Gold cigarette (0.8 mg nicotine/cigarette) (manufacturer, Phillip Morris International), used as a comparator.

Products were accompanied by instructions for use: for the nicotine pouches, a pouch was to be placed between the upper lip and gum and kept still there for 20 min; nicotine pouches were not to be chewed during the usage and were not to be swallowed (normal swallowing saliva was allowed). Twenty minutes was selected as the product usage time to reflect the product-specific use instructions for the nicotine pouch products in this study and therefore allowed clinical characterisation of the products’ use according to this. For the Marlboro Gold cigarette, subjects were told they should puff approximately every 30 s (prioritising completion of PK sampling and questionnaires at the 2-min timepoint) and should aim to complete within 10 or 11 puffs and within 5 min. However, it was allowed to take longer to finish the cigarette if required.

### Study procedure

This study was a randomised, cross-over, open label, confinement study in 24 male and female snus and cigarette consumers, using three study products, and evaluated nicotine PK, subjective effects and product safety.

The first study visit (1) took place between days -28 and -1. This included an eligibility check, a review of health status and assessment of snus and cigarette consumption habits. Subjects were provided with smoking cessation advice and contact information for a smoking cessation support service, if they requested it.

The second visit (2) was a 5-day confinement period; subjects were admitted to the clinic on the evening of day -1 and remained in the clinic until day 5. On the evening of day -1, subjects underwent baseline assessments for clinical laboratory profile, vital signs and ECG; subjects also undertook a familiarisation session with the study products and questionnaire format. In this session, the clinical team explained how the study products were to be used, and subjects had the opportunity to see the products and packaging. An explanation of how the questionnaires were to be administered to the subjects was given. The familiarisation session did not include a product trial, and all products used in the session were not to be used in the clinical study but were retained as demonstration samples for accountability purposes. Subjects were allowed to use their own products until 10 p.m. that evening. At 10 p.m., the subjects’ own nicotine-containing products were collected by a member of the clinical team and returned upon completion on day 5. On the morning of day 1, following the pre-use assessments and confirmation of eligibility, the subjects were randomised and then administered a single pouch/single cigarette according to their randomisation sequence. Randomisation was used to minimise bias in the assignment of subjects. The products were used according to the instructions provided (detailed in “Study products” section): nicotine pouches were used for 20 min and according to standard use instructions; the cigarette was to be smoked in approximately 5 min, with puffs taken at regular intervals (approximately 30 s apart). PK sampling was carried out pre-product use and at 2, 5, 7, 15, 20, 30, 45, 60, 90 min, and 2, 4, 6, 8 h post product use start. Questionnaires were administered to the subjects at defined intervals throughout the day. Participant safety was also monitored throughout the day. After the 8-h timepoint, subjects were allowed to use the product they had been assigned that day *ad lib* until 10 p.m. Meals were served while subjects were in the research clinic: breakfast was served approximately 1 h prior to product use; lunch was served 4 h after the start of each product use; snack, dinner and evening snack were served approximately 7, 9 and 11 h post-product use, respectively. Water was allowed *ad lib* at the clinic except 30 min before product use until 1 h after product use.

Days 2, 3, 4 and 5 followed the same schedule (with the exception that eligibility and randomisation took place on day 1 only and that subjects left the clinic after completion of all 8-h assessments on day 5). On day 7 (± 1), a follow-up phone call (visit 3) was made to subjects to record any adverse events (AEs). An overview of the study procedure can be found in Fig. 1.

The focus of this study was the assessment of the clinical outcomes with the use of the two nicotine pouch products compared to cigarette. In the full clinical trial, two additional products were tested, but beyond the scope of the current study, and therefore, the study was conducted over 5 days to accommodate the 5 products.

### Study assessments

#### Pharmacokinetic assessment

To determine blood plasma nicotine concentrations after use of the study products, blood samples (approx. 4 ml/sample) were collected through an indwelling venous catheter at the time-points described above. Pre-product use sampling was carried out within 5 min prior to the product use. Plasma samples were analysed for nicotine concentration by Lablytica AB using a validated LC–MS/MS method.

#### Subjective assessment

The subjects were asked to self-assess their experience of the products following use (urge to smoke (also recorded pre-dose) and product liking) using the Products Evaluation Scale (PES) at the timepoints detailed in Tables S3 and S5 (supplementary information). Participant ratings were carried out using the 100-mm visual analogue scale (VAS) with the anchor points printed on paper. For urge to smoke: 0 mm = not at all/ no urge and 100 mm = extremely/extreme urge. For product liking (pleasantness of the product, satisfaction with the product, strength (nicotine content) of the product): 0 mm = none and 100 mm = extreme. The assessment data was entered into an electronic format by the study personnel.

#### Safety and tolerability

AEs (including serious AEs (SAEs)) were recorded from the start of the first product used until the end-of-study visit (3). Severity/intensity were graded as mild, moderate or severe, and AEs were also assessed as unlikely, possibly or probably related to the study product by the investigator. Clinically significant changes in laboratory parameters (clinical chemistry, haematology, urinalysis, pregnancy, SARS-COV-2 detection), vital signs and ECGs were also assessed throughout. A breakdown of all AE results can be found in the supplementary information (Tables S7.1 and S7.2).

### Data analyses

#### Pharmacokinetics

PK analysis was carried out on data from all subjects who had used at least one of the study products, provided an evaluable plasma nicotine concentration profile and had no AEs or protocol deviations which were judged to affect the PK output (e.g. vomiting, subject not following restrictions, wrong product given). Nicotine PK parameters (*C*_max_ (ng/ml), area under the plasma concentration–time curve (AUC)_*t*_ (AUC_0−*t*_) (h*ng/ml), *T*_max_ (min), *T*_1/2(z)_ (min), AUC_0–90_ (h*ng/ml), AUC_0–inf_ (h*ng/ml) *C*_last_ (ng/ml) were calculated by non-compartmental analysis (NCA) using Phoenix WinNonlin® software (version 8.1) (Certara, USA), and analysis was based on the actual sampling times recorded during the study. Nicotine concentrations below the lower limit of quantification (LLOQ) which occurred before *C*_max_ were treated as 0; concentrations below the LLOQ which occurred after *C*_max_ were omitted from the analysis. *C*_max_ and *T*_max_ were derived from the observed plasma nicotine concentrations. The AUC was assessed by integration of the plasma nicotine concentration v. time curve using linear interpolation for increasing plasma levels and logarithmic interpolation for decreasing plasma levels (Lin Up-Log Down method). For AUC_0−90_ calculation, if there was no actual sampling timepoint at 90 min, the plasma nicotine concentration at 90 min was determined by interpolation between the surrounding and actual sampling points (according to lin up-log down principles).

AUC_*t*_ was calculated from time 0 to the time (*t*) of the last detectable plasma concentration (last timepoint). For AUC_0–inf_, area was calculated to the last timepoint with a measurable plasma concentration, then extrapolated to infinity using the concentration in the last quantifiable sample and *λ*_*z*_. *T*½ was calculated by ln2/*λ*_*z*_. *λ*_*z*_, the first-order rate constant associated with the terminal portion of the curve, was determined by lin-log regression of the terminal elimination phase of individual plasma concentration v. time curves. Determination of *λ*_*z*_ requires identification of a sufficiently linear terminal phase (as determined by visual inspection of the lin-log plasma concentration v. time plot with the regression line) consisting of at least 3 terminal concentration values (not including *C*_max_). If this was not achieved, *λ*_*z*_ and its dependent PK parameters were not reported for that profile. In the following cases, *λ*_*z*_-dependent PK parameters were flagged in listings as potentially unreliable: *λ*_*z*_ estimation was based on a period of less than 1.0 times the resulting *T*½; the adjusted *R*^2^ value of the regression line is < 0.85; the estimated % extrapolated AUC is > 20% (AUC_0–inf_ − AUC_*t*_/AUC_0–inf_). Where plasma nicotine concentrations were above the LLOQ immediately prior to product administration (pre-dose sample), PK parameters were also calculated from baseline adjusted concentrations using a subject’s elimination rate constant (*λ*_*z*_) and observed pre-dose concentration (considered to have been collected at time 0). Baseline adjustments were calculated according to the formula: *C*(*t*)_adjusted_ = *C*(*t*)_observed_ − *C*(0)e^−λ^_*z*_^(*t*^.^)^

### Statistical analyses

The following statistical comparisons were made for AUC_*t*_ and *C*_max_: #2 v. #3, #2 v. combustible cigarette (CC), #3 v. CC. The comparison of the products, on log transformed nicotine *C*_max_ and AUC_*t*_ estimates, was performed using a linear mixed-effects repeated measurements analysis of variance model. Sequence and product were the fixed effects, period a repeated effect and subject a mixed effect. Kenward-Rogers degrees of freedom approximation were used (Kenward and Roger, 1997). Covariance structures (SAS abbreviated terminology), variance components (VC), unstructured (UN), compound symmetry (CS), autoregressive (1) (AR(1)), autoregressive moving average (1,1) (ARMA (1,1)) and banded Toeplitz (TOEP(q)), for the repeated measurements were tested, and the structure with the highest adjusted Akaike criteria was used in the final analysis. The estimated product differences were back-transformed to present the ratios of geometric least squares (LS) means and 95% confidence intervals (CIs) of each test product versus each other from the same model.

#### Subjective

PES differences between products on total and subcategory PES score at each timepoint were analysed using Wilcoxon signed rank sum tests.

## Results

### Nicotine pharmacokinetics

**Fig. 2:**
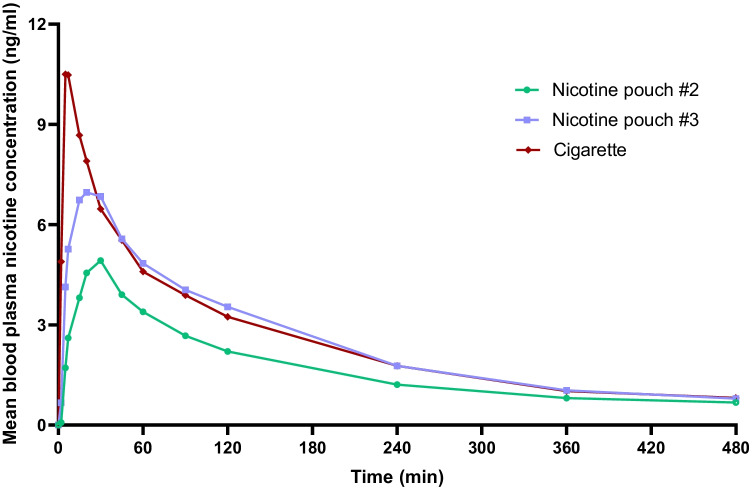
Baseline adjusted nicotine levels measured in the blood plasma of adult traditional tobacco product users during 8 h following use of a single nicotine pouch product (#2 or #3) or cigarette. For #2, *n* = 21; #3, *n* = 22; cigarette, *n* = 22. Non-baseline adjusted data plotted with error bars (standard deviation) can be found in the supplementary information (Fig. S1)

Participant blood samples were taken at defined timepoints during an 8-h period, starting at the point of single allocated product use. Cigarette use resulted in the highest average *C*_max_ recorded (11.6 ng/ml) and within the shortest time period (8.5 min) (Fig. 2, Table 1). The *C*_max_ values recorded were significantly different between the three study products (Table S2, supplementary information). The AUC_*t*_ was significantly lower for #2 nicotine pouch compared to the two other study products, which were not significantly different from one other (Table S2, supplementary information). However, the average AUC_*t*_ value for #3 nicotine pouch was observed to be slightly below that for cigarette. Additionally, *C*_max_ for the respective nicotine pouch products was reached 3 times (#2) and 2.6 times (#3) slower than with cigarette.

**Table 1:** :Baseline adjusted values for blood plasma nicotine pharmacokinetic assessments in adult traditional tobacco product users following use of a single nicotine pouch or cigarette product. *C*_*max*_ maximum observed plasma concentration, *AUC*_*t*_ area under the plasma concentration–time curve from time 0 to the time of the last sampling timepoint, *T*_*max*_ time to *C*_max_, *T*_1/2_ terminal elimination half-life, *AUC*_*0–90*_ AUC from timepoint 0–90 min, *AUC*_*0–inf*_ AUC from timepoint 0 to infinity, *C*_*last*_ observed plasma concentration at the last sampling timepoint

*Assessment (unit)*		*Nicotine pouch #2*	*Nicotine pouch #3*	*Cigarette*
	*n*	21	22	22
*C*_max_ (ng/ml)	Mean (SD)	5.154 (1.662)	7.856 (2.451)	11.60 (5.171)
	Median (min, max)	5.060 (2.66, 9.43)	6.983 (4.39, 13.5)	9.868 (4.56, 23.1)
AUC_*t*_ (h*ng/ml)	Mean (SD)	12.23 (4.996)	18.35 (8.180)	19.60 (10.75)
	Median (min, max)	11.05 (6.32, 26.1)	16.95 (8.86, 45.5)	18.24 (8.26, 54.7)
*T*_max_ (min)	Mean (SD)	26.43 (9.468)	22.092 (8.478)	8.544 (6.552)
	Median (min, max)	30 (7.98, 45)	19.98 (7.02, 31.02)	7.002 (4.998, 34.02)
*T*_1/2(*z*)_ (min)	Mean (SD)	159.36 (57.006)	146.22 (52.656)	151.74 (51.63)
	Median (min, max)	150.36 (86.4, 319.2)	135.06 (103.8, 343.2)	140.1 (101.4, 360.6)
AUC_0–90_ (h*ng/ml)	Mean (SD)	5.185 (1.611)	7.876 (2.316)	8.829 (4.012)
	Median (min, max)	4.705 (2.70, 9.80)	7.248 (4.81, 13.1)	8.141 (3.93, 19.1)
AUC_0–inf_ (h*ng/ml)	Mean (SD)	14.90 (6.984)	21.51 (11.27)	23.16 (16.52)
	Median (min, max)	13.57 (7.91, 37.7)	18.95 (11.3, 65.6)	20.08 (9.35, 87.1)
*C*_last_ (ng/ml)	Mean (SD)	0.6480 (0.2768)	0.8084 (0.4032)	0.7670 (0.7122)
	Median (min, max)	0.5670 (0.401, 1.61)	0.7221 (0.405, 2.44)	0.5717 (0.292, 3.74)

### Subjective outcomes: urge to smoke and product liking

**Fig. 3:**
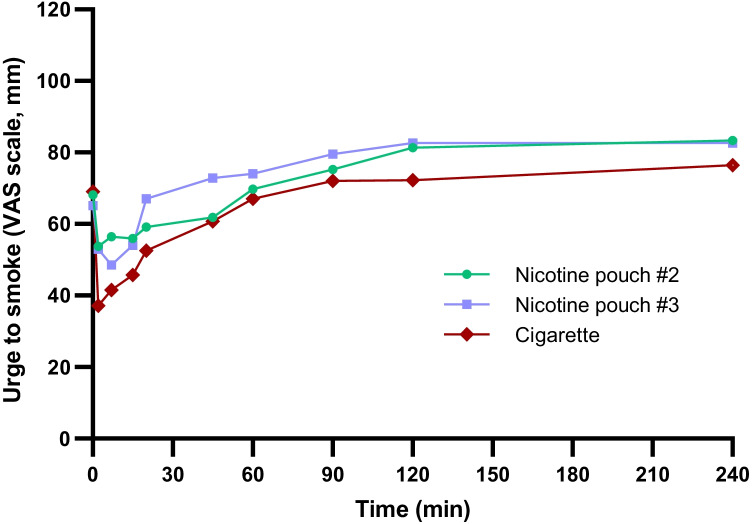
Average self-reported urge to smoke according to the 100-mm VAS scoring system self-assessed by subjects over 240 min following use of a single nicotine pouch (#2 or #3) or cigarette study products (pre-dose is plotted as 0 min). 100 = extreme urge to smoke, 0 = no urge to smoke. A lower score indicates a greater reduction in desire to smoke. For no. #2, *n* = 22; no. #3, *n* = 23; cigarette, *n* = 22. Data plotted with error bars (standard deviation) can be found in the supplementary information (Fig. S2)

Subjects were asked to assess how they felt, in terms of urge to smoke a cigarette, both pre-dose and at timepoints up to 4 h post-use (detailed in Fig. 3 and Table S3), according to a 100-mm VAS scoring system (100 = extreme urge to smoke, 0 = no urge to smoke). Pre-dose, there were no significant differences in subjects’ self-reported urge to smoke between the different products (Table S4, supplementary information). For all three products, the lowest average values, and therefore lowest average urge to smoke, were achieved at the timepoints immediately following product use (2 min and 7 min), and after this, average scores increased over time, with the highest scores observed for all three products 4 h following product use. There were few significant differences between the products at the timepoints recorded (between #2 and CC measurements at 2 min; between #3 and CC at 20 min and 45 min; between #2 and no. #3 at 90 min (Table S4, supplementary information)).

**Fig. 4:**
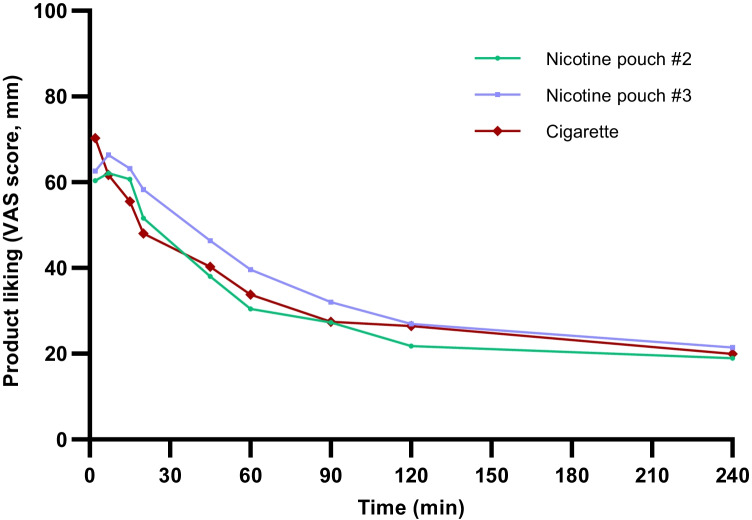
Average product liking according to the 100-mm VAS scoring system self-assessed by subjects over 2–240 min following use of a single nicotine pouch (#2 or #3) or cigarette study products. 100 = extreme, 0 = none. A higher score indicates greater product liking. For no. #2, *n* = 22; no. #3, *n* = 23; cigarette, *n* = 22. Data plotted with error bars (standard deviation) can be found in the supplementary information (Fig. S3)

Similar findings were observed for product liking, which was assessed at timepoints between 2 min product use and 4 h (Fig. 4). The highest scores were observed at the timepoints immediately following first product use (i.e. 2 min and 7 min), and following this, scores decreased with time. The highest average score was observed for cigarette; however, there were few significant differences between the products at the recorded timepoints (between #2 and CC and #3 and CC measurements at 2 min; between #3 and CC at 5 min and 20 min; between #2 and #3 at 45 min (Table S6, supplementary information).

### Safety and tolerability

No SAEs were recorded during the study. The most common AE recorded was headache, reported by two subjects on one occasion each. Both events were assessed as mild intensity, and one was assessed as possibly related to product use (cigarette) and one assessed as unlikely to be related (nicotine pouch #2). No other AE was reported by > 1 subject during the study. One AE was reported as probably related to product use: hiccups of mild intensity, occurring in association with use of nicotine pouch #2 during the ad lib use period for that product. No relevant differences between the three study products were recorded with regard to the types of AEs, AE reporting frequency, intensity or relatedness to the product.

There were no clinically significant changes from baseline measurements in mean clinical chemistry, haematology or urinalysis, overall or in any of the treatment sequences, recorded up to the end of day 5. Additionally, there were no clinically significant changes from baseline in mean systolic blood pressure, diastolic blood pressure or pulse rate, overall or with any of the study products, up to 8 h post dose, and no clinically significant changes from baseline in mean values within the ECG parameters assessed (overall or in any treatment sequence), recorded at the end of day 5. Overall, single use of the study products, followed by a short ad lib use period, was observed to be safe and well tolerated within the study population.

## Discussion

This study’s primary objective was to assess the blood plasma nicotine levels in healthy adult traditional tobacco product users following use of three study products, two nicotine pouch variants and one cigarette, administered according to a randomised single-use schedule across 5 days. The secondary assessments included in the study were self-assessed subjective effects, and safety and tolerability, assessed by the study investigator.

### PK profiles indicated oral mucosal nicotine absorption for the nicotine pouches, compared to pulmonary absorption for cigarette

Nicotine pouch products, such as those in this study, are used by inserting under the lip, and nicotine absorption into the blood occurs buccally (via the oral mucosa) (Lunell et al., 2020; Rensch et al., 2021; McEwan 2021); in the case of cigarettes, nicotine uptake is via inhalation. The PK curves following use of the study products demonstrated single nicotine peaks for each product type, from which it can be concluded that nicotine followed a single, primary route of absorption. It has been previously demonstrated that swallowing of nicotine lozenges, and therefore nicotine absorption via gastrointestinal (GI) absorption, results in very low blood plasma nicotine levels that peaks after 2–3 h (Dautzenberg et al., 2007); however, in the current study, *T*_max_ values for the nicotine pouches were in the range of 22–26 min, demonstrating a reliable correlation with end of product use (20 min). The observed PK profiles in the current study indicate oral mucosal absorption, and not via swallowing/GI absorption, as absorption via the GI route would result in less clear PK curves due to first pass elimination and the low stomach pH’s impact on uptake. Furthermore, the 8-h period over which PK measurements were taken following single product administration confirms the absence of secondary blood plasma nicotine peaks, and this, coupled with the fact that subjects were allowed to swallow any saliva during product use, demonstrates that nicotine absorption via the gastrointestinal route was very limited, if occurring at all.

### Nicotine blood plasma levels were sufficient to reduce desire to smoke following use of the nicotine pouches, and AUC values for total nicotine delivery did not exceed that for cigarette

Following respective use of the three study products, cigarette use resulted in the highest peak in blood plasma nicotine levels (*C*_max_ (11.60 ng/ml vs 5.15 ng/ml #2) and 7.856 ng/ml (#3)), and further to this, average AUC values for two nicotine pouch products did not exceed that for cigarette. Peak nicotine plasma levels were additionally reached much later following nicotine pouch use (26 min for #2 (5.8 mg nicotine/pouch); 22 min for #3 (10.1 mg nicotine/pouch)) compared to cigarette (8.5 min). This slower nicotine *T*_max_ timeframe for nicotine pouches is generally consistent with other studies on nicotine pouch product PK profiles (Lunell et al., 2020; Rensch et al., 2021; McEwan et al., 2021); however, although not directly comparable, there is some variation in nicotine *T*_max_ between commercially available nicotine pouch brands. For example, *T*_max_ for the On! brand was reported by Rensch et al. (2021) to be in the range of 30–35 min, and Lunell et al. (2020) described a 59–66-min range for Zyn product variants, with *C*_max_ levels correlating with pouch nicotine content in both studies. However, McEwan et al. (2021) reported *T*_max_ values in the range of 60–65 min for five nicotine pouch product variants, which may be attributed to the product use duration of 60 min; the study also found that nicotine bioavailability did not correlate with pouch nicotine content. In addition to differing study designs, for example, duration of product use, differences between the findings of the studies may be attributed to the different substrate matrices in which nicotine is present within the pouches and other variations between products, such as pouch material, moisture content and pH, which may affect nicotine release (Aldeek 2021; McEwan 2021) and therefore absorption in the buccal cavity during use (Pickworth et al., 2014).

Despite significant differences in nicotine *C*_max_ between the three study products and significantly lower AUC levels for the #2 nicotine pouch product, the slower and lower levels of nicotine in the blood following nicotine pouch use were effective in significantly reducing the subjects’ self-reported urge to smoke, with few significant differences across the urge to smoke profiles for all three products across the 4-h self-assessment period. These data provide valuable information on the nicotine PK characteristics and subjective effects of nicotine pouches directly compared to cigarette smoking, which is currently limited within the published scientific literature and provides valuable evidence that nicotine pouches have the potential to offer adult smokers a satisfactory level of nicotine delivery as an alternative to smoking cigarettes. Despite cigarette scoring the highest for product liking upon first use of the study products as may be expected, the nicotine pouch products achieved a comparable product liking profile to cigarette across the timepoints assessed, again indicating their potential as a highly acceptable alternative for adult smokers which may be potentially acceptable switching products for adult smokers who would otherwise continue to smoke.

In understanding the potential abuse liability of a nicotine product, a combination of nicotine pharmacokinetics, subjective effects and behavioural responses, relative to a comparator product with known abuse liability, can be informative. Based on the pharmacokinetic outcomes reported here, which demonstrate lower and slower levels of nicotine delivery compared to cigarettes, coupled with the absence of the hand-to-mouth behaviour associated with smoking, and the reported subjective response data, it is likely that based on the outcomes generated under the study conditions, the assessed nicotine pouches may have lower abuse potential than cigarettes for adult smokers (Vansickel et al., 2022). This is consistent with the findings and conclusions reported (i.e. slower speed to *T*_max_, lower magnitude of *C*_max_ and positive subjective effects relative to their own brand cigarettes) in a recently published nicotine pouch clinical study conducted by Rensch et al. (2021).

### The role of nicotine pouches in tobacco harm reduction

THR involves providing adult smokers, who would otherwise continue to smoke, with alternative nicotine products which offer reduced exposure to toxicants but can also achieve satisfactory levels of nicotine delivery. This study demonstrated that the assessed nicotine pouches achieved blood plasma nicotine levels in the subjects which were able to effectively reduce urge to smoke following use and maintain a similar urge to smoke profile to cigarette in the 4 h following product use, highlighting their potential as an acceptable alternative to cigarette smoking. However, it is recognised that nicotine delivered by such alternatives should not exceed that of cigarette; it should be sufficient to satisfy adult smokers to aid their transition away from cigarettes (Institute of Medicine, 2012). Whilst the AUC_*t*_ for the two nicotine pouches used in this study were lower than with cigarette, this was not significantly lower for the #3 product. However, the slower time to, and lower, *C*_max_ for both nicotine pouches, compared to cigarette, suggests they likely have lower abuse liability and addictive potential for current adult smokers compared to continued cigarette smoking (Rensch et al., 2021). Two nicotine pouches, of different nicotine strengths (#2: 5.8 mg nicotine/pouch and #3: 10.1 mg nicotine/pouch) were used in this study. Total (baseline adjusted) nicotine blood plasma levels correlated with the nicotine content of the respective pouches, but both products demonstrated comparable reductions in urge to smoke and product liking profiles to the cigarette, suggesting blood plasma nicotine levels are not the only contributor to product satisfaction. Offering adult smokers choices via a range of harm reduction products, in terms of nicotine content as within this study, but also flavourings and product types, may increase potential for more current adult smokers to transition away from cigarettes. It has, however, been evidenced that flavours alone may not have a great influence on subjective effects in adult nicotine pouch users in clinical assessment settings (Rensch et al., 2021), emphasising the multifactorial importance of several characteristics to realise the full THR potential of these products. The two nicotine pouches used in this study additionally demonstrated good short-term safety and tolerability profiles, and no SAEs were observed during the study. The one mild AE deemed as associated with ad lib use of the #2 nicotine pouch, hiccups, is a transient, self-limiting, known and commonly observed effect of nicotine product use (Tønnesen et al., 2012; Russell et al., 1977).

Satisfactory nicotine delivery following nicotine pouch use, as demonstrated in this study, is accompanied by reduced exposure potential to tobacco-associated toxicants during use of this product category (Azzopardi 2021). Firstly, as the products contain only high-quality pharmaceutical grade nicotine derived from tobacco leaf, and not the leaf itself, levels of associated compounds such as TSNAs and PAHs are substantially reduced (Azzopardi 2021; Bishop et al., 2020). The absence of combustion leads, again, to further substantial reductions in the presence of associated (inhaled) toxicants, which leads to reduced exposure for the adult smoker. Further to this, the nicotine pouches used in this study contain high-quality ingredients which have undergone rigorous and relevant toxicological risk assessments to determine their suitability for use. Recent analyses have demonstrated that compared to tobacco leaf-containing snus, four Lyft nicotine pouch variants possessed substantially lowered toxicant profiles, comparable to those of the medically licensed NRTs included in the study, as well as the lowest toxicant levels when compared to HPHC data for cigarette, heated tobacco and e-cigarette products (Azzopardi 2021). These findings translate to reductions in toxicological effects of nicotine pouch products compared to cigarette smoke extracts in vitro(Bishop et al., 2020). Whilst the current evidence in the scientific literature demonstrates that the nicotine pouch products have an important role to play in making a meaningful contribution to tobacco harm reduction, through their chemical compositions, pre-clinical toxicology and potential as a satisfactory nicotine delivery alternative for adult smokers, these studies are still limited. This can be attributed to the relative nascency of nicotine pouches; however, to further substantiate their THR potential and confirm their position on a relative risk scale, more studies in the areas detailed are needed.

### Study limitations and future direction

The work presented in this paper has a number of limitations, and the results should be considered within this context. This study provides valuable information on the PK, subjective and safety profiles of two commercially available nicotine pouch products, compared directly to cigarette. Further studies of this kind, comparing other commercially available nicotine pouches to cigarette, would be valuable to further substantiate the findings here that nicotine pouches offer a satisfactory level of nicotine delivery to adult smokers and therefore have an important role to play in THR strategies. As different product variations within the category of high-quality nicotine pouches contain nicotine within different substrate matrices, there is a need to further understand how this affects nicotine PK profiles, and in relation to cigarette. Therefore, the findings of this study may not be generalisable to the nicotine pouch product category.

The sample size used in this study was relatively small, although the number of subjects within the study was deemed suitable for the assessments and statistical analyses carried out. Additionally, the randomisation of subjects across the 5 days to the study products, as opposed to assessment of each product in parallel, supported the sample size used. Only one duration of product use (20 min) was used in this study; however, different product use times may affect the nicotine PK profile.

Although this study did not include analysis of biomarkers of exposure (BoE) following use of the products, there is evidence that substantial reductions in HPHCs in the aerosols of other non-combustible nicotine products, including e-cigarettes and heated tobacco products, compared to cigarette, translate to substantial reductions in BoE detected in clinical studies (Morris et al., 2021; Tran et al., 2020; Lüdicke et al., 2016). Based on the reduced toxicant levels reported in nicotine pouches (Azzopardi 2021), it may be inferred that this would also be the case for this product category. Chemical (HPHC) characterisation and pre-clinical assessment of the nicotine pouch products used in this study would provide additional evidence on their THR potential and will be published in future papers. The study design also had the advantage of direct comparison of PK and subjective effects following the controlled use of single study products; however, it may be beneficial to make these assessments under ad libuse to further characterise the measured effects under more realistic product use conditions. However, this may still not fully represent real-world use; further to this, longer term safety and tolerability needs to be assessed for these products.

## Conclusions

This study was the first clinical study, to our knowledge, to directly compare the PK and subjective characteristics of the two commercially available ZoneX #2 and #3 nicotine pouch products to cigarette. The time taken to reach peak blood plasma nicotine was slower, and systemic nicotine levels lower, than with cigarette following use of the nicotine pouches; however, the assessed nicotine pouches were still able to offer subjects a satisfactory level of nicotine delivery (measured through urge to smoke and product liking) at two different product nicotine strengths, whilst not exceeding nicotine delivery compared to smoking a cigarette. The study also highlighted that the nicotine delivery following nicotine use is via a single primary route (i.e. buccal cavity/oral mucosal) and demonstrated that nicotine pouches have a good short-term safety and tolerability profile. Overall, the study has demonstrated that nicotine pouches have an important role to play in THR strategies for adult smokers who would otherwise continue to smoke cigarettes, through efficient delivery of nicotine to the blood coupled with favourable product liking and reductions in urge to smoke scores.

Supplementary information.

## Supplementary information

Below is the link to the electronic supplementary material.

Supplementary file1 (RTF 4553 KB)

## Abbreviations

AE: Adverse event
AR(1): Autoregressive(1) (SAS abbreviated term)
ARMA(1,1): Autoregressive moving average(1,1) (SAS abbreviated term)
AUC: Area under the plasma concentration–time curve
AUC_*t*_: AUC from 0 to the time of the last sampling timepoint, i.e. time 0-last
AUC_0–90_: AUC for timepoints 0–90 min
AUC_0–__inf_: AUC for timepoints 0–infinity
bpm: Beats per minute
BMI: Body mass index
CI: Confidence interval
C_last_: Observed nicotine plasma concentration at the last sampling timepoint
C_max_: Maximum observed plasma concentration
CS: Compound symmetry (SAS abbreviated term)
ECG: Electrocardiogram
GCP: Good clinical practice
Hb: Haemoglobin
HIV: Human immunodeficiency virus
ICH: International Council for Harmonisation of Technical Requirements for Pharmaceuticals for Human Use
LLOQ: Lower limit of quantification
LS: Least squares (mean)
mmHg: Millimetre mercury (unit for blood pressure measurement)
NCA: Non-compartmental analysis
NGP: Next generation product
OND: Oral nicotine delivery
PAH: Polycyclic aromatic hydrocarbon
PES: Product evaluation scale
PK: Pharmacokinetic
SAE: Serious adverse event
SAS: Statistical Analysis System (software)
SD: Standard deviation
SERA: Swedish Ethical Review Authority
THR: Tobacco harm reduction
*T*_max_: Time to *C*_max_
TOEPq: Banded Toeplitz (SAS abbreviated term)
TSNA: Tobacco specific nitrosamine
T_1/2_: Terminal elimination half-life
UN: Unstructured (SAS abbreviated term)
VAS: Visual analogue scale
VC: Variance components (SAS abbreviated term)
