# Supplementary material for: A randomised, open-label, cross-over clinical study to evaluate the pharmacokinetic, pharmacodynamic and safety and tolerability profiles of tobacco-free oral nicotine pouches relative to cigarettes
Source: Psychopharmacology (Berl). 2022 Jun 23;239(9):2931–43. doi: 10.1007/s00213-022-06178-6 (PMC9217727; doi:10.1007/s00213-022-06178-6)
Supplement: Supplementary file 1 — Supplementary file1 (RTF 4553 KB) [file 213_2022_6178_MOESM1_ESM.rtf]

A randomised, open-label, cross-over clinical study to evaluate the pharmacokinetic, pharmacodynamic and safety and tolerability profiles of tobacco-free oral nicotine pouches relative to cigarettes 
Fiona Chapman*1a, Simon McDermott*1, Kathryn Rudd1, Victoria Taverner1, Matthew Stevenson1, Nveed Chaudhary1, Kerstin Reichmann1, Joseph Thompson1, Thomas Nahde2, Grant O'Connell1
1Imperial Brands PLC, 121 Winterstoke Road, BS3 2LL, Bristol, UK
2Reemtsma Cigarettenfabriken GmbH, an Imperial Brands PLC Company, Albert-Einstein-Ring-7, D-22761, Hamburg, Germany 
*Joint first author
aCorresponding author (email: Fiona.Chapman@uk.imptob.com; Telephone: +44 (0) 117 332 2523)


Supplementary information
Table S1: Pharmacokinetic analysis dataset – measured values of blood plasma nicotine at specified timepoints up to 8h following single product use. BLQ: Below lower limit of quantification; ULQ: Above upper limit of quantification; NC: Not calculated – number of non-missing observations <3; n: Number of subjects.
Blood plasma nicotine concentration (ng/ml)	Nicotine pouch #2	Nicotine pouch #3	Cigarette	
Pre-dose	n/BLQ/ULQ	21/9/0	22/8/0	23/1/0	
	Mean
(SD)	1.351
(2.545)	1.527
(4.220)	1.795
(2.068)	
	Median
(min, max)	0.6010
(0.00, 11.4)	0.5500
(0.00, 20.2)	1.310
(0.00, 10.5)	
2min	n/BLQ/ULQ	21/6/0	22/4/0	22/0/0	
	Mean
(SD)	1.414
(2.335)	2.217
(4.093)	6.727
(3.816)	
	Median
(min, max)	0.6270
(0.00, 10.5)	1.310
(0.00, 19.9)	5.865
(1.69, 18.9)	
5min	n/BLQ/ULQ	21/1/0	23/0/0	21/0/0	
	Mean
(SD)	3.041
(2.667)	5.521
(5.710)	12.40
(6.122)	
	Median
(min, max)	2.980
(0.00, 12.3)	4.620
(0.609, 29.9)	10.30
(4.53, 24.9)	
7min	n/BLQ/ULQ	21/0/0	23/0/0	22/0/0	
	Mean
(SD)	3.925
(3.085)	6.617
(5.009)	12.28
(5.286)	
	Median
(min, max)	3.460
(0.594, 14.6)	5.830
(1.03, 26.3)	10.50
(5.10, 26.9)	
15min	n/BLQ/ULQ	22/0/0	22/0/0	22/0/0	
	Mean
(SD)	4.946
(2.869)	8.200
(5.367)	10.42
(5.171)	
	Median
(min, max)	4.690
(1.70, 14.9)	7.610
(2.72, 29.3)	9.555
(4.75, 28.0)	
20min	n/BLQ/ULQ	22/0/0	22/0/0	21/0/0	
	Mean
(SD)	5.691
(3.284)	8.402
(5.216)	9.607
(4.976)	
	Median
(min, max)	4.790
(2.54, 16.7)	6.850
(3.00, 28.5)	8.460
(4.75, 26.1)	
30min	n/BLQ/ULQ	22/0/0	23/0/0	22/0/0	
	Mean
(SD)	6.005
(3.299)	8.384
(5.432)	8.108
(4.124)	
	Median
(min, max)	5.740
(2.87, 17.7)	7.390
(4.39, 30.8)	7.355
(3.77, 21.4)	
45min	n/BLQ/ULQ	22/0/0	23/0/0	22/0/0	
	Mean
(SD)	4.955
(2.790)	7.270
(5.041)	7.078
(4.286)	
	Median
(min, max)	4.025
(2.56, 14.5)	5.500
(3.44, 26.8)	6.000
(2.87, 22.0)	
60min	n/BLQ/ULQ	22/0/0	23/0/0	22/0/0	
	Mean
(SD)	4.390
(2.974)	6.509
(4.993)	6.043
(3.565)	
	Median
(min, max)	3.720
(2.21, 15.8)	5.260
(2.52, 26.1)	4.930
(2.98, 19.2)	
90min	n/BLQ/ULQ	22/0/0	23/0/0	22/0/0	
	Mean
(SD)	3.595
(2.459)	5.563
(5.065)	5.174
(3.290)	
	Median
(min, max)	2.850
(1.93, 13.3)	4.220
(2.03, 26.2)	4.175
(2.34, 17.7)	
120min	n/BLQ/ULQ	22/0/0	23/0/0	22/0/0	
	Mean
(SD)	3.049
(2.488)	4.843
(5.290)	4.386
(3.033)	
	Median
(min, max)	2.275
(1.47, 12.8)	3.390
(1.82, 27.6)	3.520
(1.86, 16.5)	
240min	n/BLQ/ULQ	22/0/0	23/0/0	22/0/0	
	Mean
(SD)	1.782
(1.870)	2.682
(3.191)	2.505
(2.356)	
	Median
(min, max)	1.250
(0.679, 9.59)	1.730
(0.847, 16.4)	1.830
(1.07, 12.5)	
360min	n/BLQ/ULQ	22/5/0	23/5/0	22/0/0	
	Mean
(SD)	1.036
(1.640)	1.436
(2.623)	1.498
(1.940)	
	Median
(min, max)	0.6800
(0.00, 7.93)	0.8830
(0.00, 13.0)	1.080
(0.525, 9.97)	
480min	n/BLQ/ULQ	22/12/0	23/9/0	22/6/0	
	Mean
(SD)	NC	0.9257
(2.061)	0.9065
(1.624)	
	Median
(min, max)	NC
(0.00, 5.65)	0.6360
(0.00, 10.1)	0.5990
(0.00, 7.91)	


Table S2: Statistical analyses between study products (nicotine pouches, #2 and #3, cigarette (CC) for the pharmacokinetic (PK) parameters, Cmax, area under the plasma concentration time curve (AUC)0-last and AUC0-inf. CI: Confidence interval; LS: Least squares mean.
PK variable	Test product	Reference product	95% CI lower bound	Ratio of geometric LS means	95% CI upper bound	Unadjusted p-value	Tukey-Kramer adjusted p-value	
Cmax
Baseline adjusted (ng/ml)	#2	#3	0.5444	0.6280	0.7243	<0.0001*	<0.0001*	
	#2	CC	0.4000	0.4608	0.5307	<0.0001*	<0.0001*	
	#3	CC	0.6374	0.7337	0.8446	<0.0001*	<0.0003*	
AUCt baseline adjusted (h*ng/ml)	#2	#3	0.5491	0.6389	0.7434	<0.0001*	<0.0001*	
	#2	CC	0.5550	0.6448	0.7491	<0.0001*	<0.0001*	
	#3	CC	0.8693	1.0093	1.1718	0.9024	0.9999	
AUC0-inf (h*ng/ml)	#2	#3	0.5706	0.6608	0.7653	<0.0001*	<0.0001*	
	#2	CC	0.5905	0.6827	0.7894	<0.0001*	<0.0001*	
	#3	CC	0.8940	1.0332	1.1941	0.6545	0.9914	


Table S3: Subjects' self-assessed (visual analogue score) urge to smoke scores pre-dose and in the 4h following use of one of three study products (nicotine pouches, #2 or #3 and cigarette). n: Number of subjects evaluated; SD: Standard deviation.
Product	Nicotine pouch #2	Nicotine pouch #3	Cigarette	
Time	n	22	23	22	
Pre-dose	Mean
(SD)	68.15
(17.94)	65.17
(19.48)	69.03
(18.35)	
	Median
(min, max)	74.40
(31.9, 93.1)	64.60
(31.5, 97.0)	71.80
(41.8, 100.0)	
2min	Mean
(SD)	53.76
(25.35)	52.90
(32.45)	37.15
(30.79)	
	Median
(min, max)	57.00
(3.0, 91.0)	57.45
(0.0, 99.0)	34.60
(0.0, 84.0)	
7min	Mean
(SD)	56.42
(25.33)	48.58
(31.62)	41.50
(29.51)	
	Median
(min, max)	55.50
(2.0, 95.0)	58.50
(0.0, 93.0)	46.50
(0.0, 86.8)	
15min	Mean
(SD)	55.94
(28.80)	54.08
(27.82)	45.78
(32.56)	
	Median
(min, max)	66.00
(4.0, 98.0)	63.50
(0.5, 89.5)	52.00
(0.0, 100.0)	
20min	Mean
(SD)	59.13
(32.14)	67.06
(22.51)	52.56
(30.81)	
	Median
(min, max)	68.00
(0.0, 100.0)	72.00
(13.5, 98.0)	58.25
(0.0, 99.0)	
45min	Mean
(SD)	61.88
(32.30)	72.89
(24.16)	60.77
(29.62)	
	Median
(min, max)	66.00
(0.0, 100.0)	80.50
(0.0, 100.0)	60.60
(0.0, 100.0)	
60min	Mean
(SD)	69.78
(23.55)	74.03
(25.48)	67.08
(26.78)	
	Median
(min, max)	70.30
(21.0, 100.0)	80.00
(1.0, 100.0)	71.50
(5.0, 99.2)	
90min	Mean
(SD)	75.20
(19.67)	79.59
(16.50)	72.03
(24.53)	
	Median
(min, max)	77.50
(34.0, 100.0)	83.00
(40.0, 100.0)	74.10
(1.0, 100.0)	
120min	Mean
(SD)	81.31
(16.77)	82.65
(17.36)	72.20
(29.28)	
	Median
(min, max)	84.50
(39.0, 100.0)	88.50
(37.0, 100.0)	82.60
(0.0, 100.0)	
240min	Mean
(SD)	83.33
(16.65)	82.63
(16.43)	76.48
(30.15)	
	Median
(min, max)	87.00
(35.0, 100.0)	85.00
(47.0, 100.0)	86.50
(0.0, 100.0)	


Table S4: Statistical analyses between study products (nicotine pouches, #2 and #3, cigarette (CC) for subjects' self-scored (visual analogue scale) urge to smoke pre-dose and in the 4h following use of a single product. CI: Confidence interval. *p<0.05.
Assessment timepoint	Test product	Reference product	95% CI lower bound	Estimated difference	95% CI upper bound	p-value	
Pre-dose	#2	#3	-12	10.05	57.5	0.3843	
	#2	CC	-21.5	-4.5	27.6	0.8631	
	#3	CC	-62.9	-32	10	0.2068	
2	#2	#3	-4.3	0.1	10	0.9866	
	#2	CC	0	12	33.2	0.0473*	
	#3	CC	-0.5	18.75	39	0.0843	
7	#2	#3	-2.5	4.35	23	0.0576	
	#2	CC	-4	10	33	0.1057	
	#3	CC	-5	1.75	25.5	0.3516	
15	#2	#3	-1	4.25	18.5	0.2515	
	#2	CC	-9.5	1	50	0.4173	
	#3	CC	-15	11	37.5	0.3884	
20	#2	#3	-8	-4	3	0.0948	
	#2	CC	-7	3	20.5	0.3790	
	#3	CC	-2	15.25	27	0.0258*	
45	#2	#3	-13.5	-2.75	3.8	0.2122	
	#2	CC	-6.9	4	10	0.6624	
	#3	CC	-0.8	4.2	19	0.0209*	
60	#2	#3	-7	-1	5.5	0.4485	
	#2	CC	-8.4	-1	8	0.8140	
	#3	CC	0	4.5	19.3	0.1384	
90	#2	#3	-9	-3.7	0	0.0070*	
	#2	CC	-6	-0.65	6	0.9466	
	#3	CC	-2	2.8	12.5	0.1536	
120	#2	#3	-3.5	-0.5	3	0.4920	
	#2	CC	-2	0.5	14	0.2898	
	#3	CC	0	1.45	16	0.0579	
240	#2	#3	-2	0.25	2	0.8273	
	#2	CC	-1.9	0.25	6	0.4806	
	#3	CC	-1.5	-0.2	9.5	0.4692	


Table S5: Subjects' self-assessed (visual analogue score) product liking scores in the 4h following use of one of three study products (nicotine pouches, #2 or #3 and cigarette). n: Number of subjects evaluated; SD: Standard deviation.
Time post-dose (min)	#2	#3	Cigarette	
	n	22	23	22	
2	Mean
(SD)	60.39
(11.91)	62.65
(17.86)	70.32
(17.62)	
	Median
(min, max)	61.60
(29.7, 76.3)	61.10
(21.8, 89.5)	75.60
(20.5, 90.0)	
7	Mean
(SD)	62.13
(16.58)	66.40
(16.88)	61.70
(19.55)	
	Median
(min, max)	63.00
(24.8, 90.1)	64.10
(36.2, 92.2)	66.35
(5.7, 85.0)	
15	Mean
(SD)	60.70
(18.72)	63.22
(19.72)	55.52
(17.45)	
	Median
(min, max)	62.10
(22.0, 92.3)	61.30
(25.7, 94.4)	50.60
(16.5, 82.7)	
20	Mean
(SD)	51.63
(23.35)	58.25
(22.12)	48.05
(22.20)	
	Median
(min, max)	53.10
(5.8, 92.7)	54.70
(12.5, 92.0)	49.75
(0.2, 80.0)	
45	Mean
(SD)	38.02
(27.30)	46.37
(24.69)	40.30
(21.82)	
	Median
(min, max)	39.85
(0.0, 85.4)	49.00
(0.7, 79.7)	40.70
(0.0, 72.8)	
60	Mean
(SD)	30.47
(25.55)	39.61
(24.06)	33.80
(23.95)	
	Median
(min, max)	26.85
(0.0, 76.9)	39.60
(0.3, 76.8)	33.65
(0.3, 91.7)	
90	Mean
(SD)	27.30
(24.53)	32.02
(21.73)	27.48
(21.37)	
	Median
(min, max)	22.65
(0.0, 77.2)	33.20
(0.3, 75.7)	25.85
(0.0, 71.7)	
120	Mean
(SD)	21.77
(21.81)	26.99
(21.44)	26.42
(23.03)	
	Median
(min, max)	17.60
(0.0, 75.6)	27.80
(0.0, 76.3)	23.00
(0.0, 71.0)	
240	Mean
(SD)	18.99
(18.82)	21.48
(20.48)	19.97
(18.44)	
	Median
(min, max)	16.35
(0.0, 74.8)	19.30
(0.0, 74.0)	20.60
(0.0, 59.8)	


Table S6: Statistical analyses between study products (nicotine pouches, #2 and #3, cigarette (CC) for subjects' self-scored (visual analogue scale) product liking in the 4h following use of a single product. CI: Confidence interval. *p<0.05.
Assessment timepoint	Test product	Reference product	95% CI lower bound	Estimated difference	95% CI upper bound	p-value	
2	#2	#3	-10	5.55	23.6	0.7756	
	#2	CC	-59	-27.25	-5	0.0057*	
	#3	CC	-60	-21.75	5	0.0467*	
7	#2	#3	-23.5	-6.35	6.9	0.1528	
	#2	CC	-17.5	-3.7	34.9	0.7656	
	#3	CC	0.5	13.5	30.5	0.1528	
15	#2	#3	-29.2	-6.7	6.4	0.2796	
	#2	CC	-5.1	18	37	0.0820	
	#3	CC	-11.5	23.1	59	0.0359*	
20	#2	#3	-50	-8.55	5.8	0.0950	
	#2	CC	-18	5	37	0.3884	
	#3	CC	8.2	37.35	78.5	0.0392*	
45	#2	#3	-45.3	-13.95	-0.5	0.0044*	
	#2	CC	-20.5	2.65	24.5	0.9875	
	#3	CC	-12	8	48.5	0.2538	
60	#2	#3	-46.2	-30.75	0.3	0.0074*	
	#2	CC	-24.1	-2.25	20.6	0.7181	
	#3	CC	-13	11.45	35.2	0.2921	
90	#2	#3	-25.5	-9.5	4.5	0.1290	
	#2	CC	-8.5	2.45	24	0.7562	
	#3	CC	-8	12.25	40.7	0.2395	
120	#2	#3	-38.5	-1.2	5.5	0.1871	
	#2	CC	-33	1.25	15	0.4787	
	#3	CC	-8	-0.25	29	0.8934	
240	#2	#3	-18	-2	2.3	0.3247	
	#2	CC	-3	0	13.3	0.9765	
	#3	CC	-4.8	0.25	7.7	0.6809	


Table S7.1: Overview of adverse events recorded during the study. n: Number of subjects; m: Number of events. Percentages are based on the number of subjects included in the analysis group. 
	Nicotine pouch #2	Nicotine pouch #3	Cigarette	Follow-up	
n	22	23	22	22	
	n (%)	m	n (%)	m	n (%)	m	n (%)	m	
Any AE	3 (14)	3	0	0	3 (14)	3	1 (5)	1	
Any SAE	0	0	0	0	0	0	0	0	
Any AE leading to withdrawal	0	0	0	0	0	0	0	0	
Any AE leading to withdrawal from study product	0	0	0	0	0	0	0	0	
Any AE leading to death	0	0	0	0	0	0	0	0	
Causality									
Unlikely related	2 (9)	2	0	0	2 (9)	1	1 (5)	1	
Possibly related	0	0	0	0	1 (5)	0	0	0	
Probably related	1 (5)	1	0	0	0	0	0	0	
Severity									
Mild	3 (14)	3	0	0	3 (14)	1	1 (5)	1	
Moderate	0	0	0	0	0	0	0	0	
Severe	0	0	0	0	0	0	0	0	


Table S7.2: Adverse events according to system organ class (bold) and preferred term. n: Number of subjects; m: Number of events. Percentages are based on the number of subjects included in the analysis group.
	Nicotine pouch #2	Nicotine pouch #3	Cigarette	Follow-up	
n	22	23	22	22	
	n (%)	m	n (%)	m	n (%)	m	n (%)	m	
Gastrointestinal disorders	 0	0	0	0	0	0	0	0	
Aphthous ulcer	0	0	0	0	0	0	0	0	
Skin and subcutaneous tissue disorders	0	0	0	0	0	0	0	0	
Pruritus	0	0	0	0	0	0	0	0	
Vascular disorders	1 (5)	1	0	0	0	0	0	0	
Thrombophlebitis	1 (5)	1	0	0	0	0	0	0	
Musculoskeletal and connective tissue disorders	0	0	0	0	1 (5)	1	1 (5)	1	
Arthralgia	0	0	0	0	1 (5)	1	0	0	
Myalgia	0	0	0	0	0	0	1 (5)	1	
Nervous system disorders	1 (5)	1	0	0	1 (5)	1	0	0	
Headache	1 (5)	1	0	0	1 (5)	1	0	0	
Respiratory, thoracic and mediastinal disorders	1 (5)	1	0	0	1 (5)	1	0	0	
Epistaxis	0	0	0	0	1 (5)	1	0	0	
Hiccups	1 (5)	1	0	0	0	0	0	0	


Fig. S1: Non-baseline adjusted nicotine levels measured in the blood plasma of adult traditional tobacco product users during 8h following use of a single nicotine pouch product (#2 or #3) or cigarette. For #2, n = 21; #3, n = 22; cigarette, n = 22. Error bars represent standard deviation.


Fig. S2: Average urge to smoke according to the 100mm VAS scoring system self-assessed by subjects over 240min following use of a single nicotine pouch (#2 or #3) or cigarette study products (pre-dose is plotted as 0min). 100 = extreme urge to smoke, 0 = no urge to smoke. A lower score indicates a greater reduction in desire to smoke. For #2, n = 22; #3, n = 23; cigarette, n = 22. Error bars represent standard deviation.


Fig. S3: Average product liking according to the 100mm VAS scoring system self-assessed by subjects over 2-240min following use of a single nicotine pouch (#2 or #3) or cigarette study products. 100 = extreme, 0 = none. A higher score indicates greater product liking. For #2, n = 22; #3, n = 23; cigarette, n = 22. Error bars represent standard deviation.
